# HEALTH DISPARITIES, EQUITY, AND ETHICAL RESPONSIBILITY OF GERONTOLOGISTS TO PROVIDE CULTURALLY CONCORDANT CARE

**DOI:** 10.1093/geroni/igae098.1404

**Published:** 2024-12-31

**Authors:** Karen Bullock, Kim Stansbury, Terrance Ruth

**Affiliations:** Boston College, Chestnut Hill, Massachusetts, United States; North Carolina State University, Raleigh, North Carolina, United States; NC STATE UNIVERSITY, Raleigh, North Carolina, United States

## Abstract

• The medical advance of today make it possible for people to live longer with serious illnesses. Many older adults will reach a point where medical technology may be able to keep them alive, but neither restore nor improve their health outcomes. Public opinion polls reveal that most people would rather be home than in a hospital or nursing home when dealing with cancer. However, many racially diverse patients and families do not prefer to transition to home with hospice and palliative care. This leaves many underrepresented serious illness care patients and families to face difficult choices about the kind of care they prefer at end-of-life. Thus, despite the capability of modern medicine to ease most pain and suffering for patients with incurable disease, the existence of good models of holistic medical interventions lack culturally-specific care. This presentation examines the ethical issues and barriers to providing equitable serious illness care. Participants will be able to (1) discuss advance care planning as a tool for effective communication and treatment planning, (2) facilitate a dialogue about ethical, professional, and personal issues relative to oncology treatment decisions, (3) describes strategies for preparing practitioners to address disparities in service delivery.

